# The relationship between agricultural biodiversity, dietary diversity, household food security, and stunting of children in rural Kenya

**DOI:** 10.1002/fsn3.387

**Published:** 2016-05-20

**Authors:** Florence K. M'Kaibi, Nelia P. Steyn, Sophie A. Ochola, Lissane Du Plessis

**Affiliations:** ^1^ Stellenbosch UniversityCape TownSouth Africa; ^2^ Division of Human NutritionDepartment of Human BiologyUniversity of Cape TownAnzio RoadCape TownSouth Africa; ^3^ Department of Foods, Nutrition and DieteticsKenyatta UniversityNairobiKenya; ^4^ Division of Human NutritionStellenbosch UniversityCape TownSouth Africa; ^5^ Present address: Kenya Technical Trainers CollegeNairobiKenya

**Keywords:** Agricultural biodiversity, dietary diversity, household food security, stunting

## Abstract

The study was to determine the role of Dietary diversity (DD), household food security (HFS), and agricultural biodiversity (AB) on stunted growth in children. Two cross‐sectional studies were undertaken 6 months apart. Interviews were done with mothers/caregivers and anthropometric measurements of children 24–59 months old. HFS was assessed by household food insecurity access scale (HFIAS). A repeated 24‐h recall was used to calculate a dietary diversity score (DDS). Agricultural biodiversity (AB) was calculated by counting the number of edible plants and animals. The study was undertaken in resource‐poor households in two rural areas in Kenya. Mothers/Care givers and household with children of 24–59 months of age were the main subjects. The prevalence of underweight [WAZ <−2SD] ranged between 16.7% and 21.6% and stunting [HAZ <−2SD] from 26.3% to 34.7%. Mean DDS ranged from 2.9 to 3.7 and HFIAS ranged from 9.3 to 16.2. AB was between 6.6 and 7.2 items. Households with and without children with stunted growth were significantly different in DDS (*P* = 0.047) after the rainy season and HFIAS (*P* = 0.009) in the dry season, but not with AB score (*P* = 0.486). The mean AB for households with children with stunted growth were lower at 6.8, compared to 7.0 for those with normal growth, however, the difference was insignificant. Data indicate that households with children with stunted growth and those without are significantly different in DDS and HFIAS but not with AB. This suggests some potential in using DDS and HFIAS as proxy measures for stunting.

## Introduction

Despite strides made to reduce global hunger through, among others, increased cereal production, vulnerable people on a global scale are still hungry. The availability of cheap cereal foods has coincided with the erosion of agricultural biodiversity and reduction in dietary diversity (Frison et al. 2005). The United Nations General Assembly declared 2010 to be the International Year of Biodiversity. This provided an unprecedented opportunity to raise awareness and promote the role that agricultural biodiversity plays in the lives of people, particularly those in low income countries (Food and Agriculture Organization (FAO) 2010) Agricultural biodiversity is the basis of the food chain which contributes to food and livelihood security, especially in the developing countries which highly depend on own food supply and food‐based strategies rooted in the sustainable use of biological resources to improve local diets (Wispelwey and Deckelbaum 2009).

A study in Kenya by Ekesa et al. (2008) showed a strong relationship between agricultural biodiversity and dietary diversity. The findings indicated that almost 50% of changes in dietary intake of preschool children were due to agricultural biodiversity. This implies that improving agricultural biodiversity could improve dietary diversity in this group which in turn could lead to an improvement in nutritional status (Ekesa et al. 2008). In addition, a systematic review on the contribution of edible plant and animal biodiversity to human diets by Penafiel et al. (2011) showed that there have only been a limited number of studies in this area and methods of assessing agricultural biodiversity are still uncertain. The complexity of linking agriculture and health appears to be a setback to engage in a large comprehensive study in this topic Penafiel et al. (2011) suggested a need for multidisciplinary research which incorporates appropriate biodiversity and nutritional assessment methodologies, in order to have a better understanding of dietary contributions of local food biodiversity and diets thus emphasizing the importance of this area of study.

The nutritional status of children under 5 years of age is an important indicator of household food security (Government of Kenya (GOK) 2008a). In Kenya, 1.8 million children are classified as chronically malnourished with poor breastfeeding and infant feeding practices contributing to more than 10,000 deaths per year (GOK 2008a), Reduction in undernutrition has been very slow, as trends over the past years show continuous deterioration (Food and Agriculture Organization (FAO) 2006; GOK 2007, 2008b, 2009a) Data from the Kenya Demographic and Health Survey (KDHS) 2014, however, showed that chronic malnutrition among children below 5 years had shown improvement since 2009. The prevalence of stunting and severe stunting was 26% and 8.1%, respectively. Estimates also showed that the Eastern Province recorded a high rate of stunting among children under 5 years of age (30.1% and 8.2% −2SD (Food and Agriculture Organization (FAO) 2010) and −3SD respectively) GOK 2014. In the latest national survey in Kenya, (GOK 2014) findings showed that 4% of Kenyan children under 5 years old were wasted, with 0.9% severely wasted. 11% of these children were also found to be underweight, with 2.3% being classified as severely underweight GOK 2014.

Research has shown that stunting, which is caused by long‐term deprivation of food or chronic undernutrition, has long‐term consequences. Stunting leads to delayed motor development, impaired immune function and generally affects cognitive development. Poor cognitive development may lead to diminished intellectual performance, decreased attention span, and poor academic achievement. Furthermore, stunting leads to reduced work capacity, increased risk of delivery complications in women, and diminished intellectual performance (Martorell 1999; Branca and Ferrari 2002; Victora et al. 2008). Stunting is also associated with a developmental delay and retarded achievement of the main child development milestones, such as walking. This might create an overall comparative disadvantage in an already difficult environment.

Interventions to correct growth and development are possible at least until the age of 5 years and are justified; although the extent to which catch‐up growth is possible and the long‐term implication of this remain to be clarified (Branca and Ferrari 2002). Hence, the high prevalence of stunting in Kenya needs urgent attention to reduce long‐term effects on the health of the population. The link between agricultural biodiversity and nutrition is not direct because of the complex nature of food security and nutrition. There are many arguments on how human nutrition is affected by agriculture through several pathways, of which producing sufficient food is just one (Pinstrup‐Andersen 2012).

The paucity of data on the relationship between agricultural biodiversity, dietary diversity, and food security with nutritional status, in Kenya, and other countries in sub‐Saharan Africa, prompted this study to build on the findings of Ekesa et al. (2008) and to contextualize the associations between agricultural biodiversity, dietary diversity, food security, and anthropometric data of children. This study was carried out during two different seasons, in two areas of different agricultural biodiversity based largely on differing rainfall figures. Household food security, as measured by household food insecurity access scale (HFIAS), was included as a variable in this study. The study also used a repeated 24 h recall to assess the variability in the diet in the households and added focus group discussions to validate the data collected using the a household questionnaire.

## Methods

### Sample

The required sample of 500 respondents (250 in each area) was based on an effect size of 0.4 with 90% power and a significance level of 5%. Percent stunting of 35% was the principle indicator used to determine sample size calculation. Households were randomly selected by means of random number tables. Two cross‐sectional studies were undertaken in resource‐poor households in two rural areas in Meru in the Eastern Province of Kenya, approximately 6 months apart. The two areas were Akithii and Uringu with the latter having a marginally better rainfall and geographic assets. Phase 1 of the study was conducted during the dry season and phase 2 after the rainy season. Of the intended sample of 500, 261 were finally realized from Uringu division and 264 from Akithii division. The extra 11 and 14 for Uringu and Akithii, respectively, were allowed for the purposes of compensating incomplete questionnaires. The repeated visits were not at the same households but were randomly selected during both phases. Interviews with mothers/caregivers were used to collect data from randomly selected households and anthropometric measurements were taken for children 24–59 months old. The 24–59 month age group of children was chosen because at this age, the children are expected to be eating from the family pot and therefore their nutritional status will be a reflection of the type of diet the families are consuming. Interviews and anthropometric measures were done by trained senior nutrition students and nutrition graduates from the University of Kenyatta who were trained by an experiences dietitian.

### Data collection instruments

#### Socio‐demographic questionnaire

The socio‐demographic part of the questionnaire elicited information on the socioeconomic status of the household. The questionnaire included questions on marital status, highest level of formal education for the mothers/caregivers, decision maker on the types of food bought and amount of money spent on food in the household. Other questions included: the number of people eating from one pot at least 4 days a week, the number of rooms per house (excluding bathroom/toilet), the type of toilet, and the sources of drinking water for the household. Additional questions included the fuel used for cooking, employment status of the care giver, number of contributors to the total household income, agricultural land size, and number and type of assets which have an influence on the economic status of the household which could in turn influence household food security and dietary diversity.

### Dietary diversity

Dietary diversity was measured using a repeated 24‐h recall with the mother/care giver. Only one child of age 24–59 months from each household participated in the survey. In cases where there was more than one child in this age bracket, one child was randomly selected. The 24‐h recall was administered to each household and repeated on a separate day. The 2 days were staggered with 5 days between the recalls to take care of the variations in foods eaten over the weekend and week days. No prior notice of the repeat visit was given to mothers/caretakers in case they altered their intake. To assess the differences caused by seasonality, two recalls were conducted in the rainy season and two in the dry season in each of the sampled areas per respondent. The repeated 24‐h dietary recall has been internationally used and validated (Steyn et al. 2006).

Dietary diversity was measured using unquantified data from the 24‐h recalls. The dietary diversity score (DDS) was calculated for each person based on nine food groups as recommended by Food and Agriculture Organization (FAO), (2011). The groups included (1) Cereals, roots, and tubers; (2) vitamin A‐rich fruits and vegetables; (3) nonrich vitamin A (other) fruit; (4) nonrich vitamin A (other) vegetables; (5) legumes and nuts; (6) meat, poultry, and fish; (7) fats and oils; (8) dairy; and (9) eggs. Other remaining items such as; tea, sugar, and sweets were not used in the DDS. DDS was calculated by summing the number of food groups consumed by the child as reported over the 24‐h recall period. This was done after creating food group variables for those food groups that needed to be aggregated and by creating a new variable termed as DDS. The maximum and ideal score would be a DDS of 9 since this would mean that the child had consumed from each of the 9 food groups at least once.

### Household food security

Household food security (HFS) was measured using the HFIAS developed by Coates et al. (2007) which is internationally used and validated. This assessment tool was used to estimate the prevalence of food insecurity in the area of study. It is based on the principle that the experience of food insecurity causes predictable reactions and responses that can be captured and quantified through a survey and summarized in a scale. These feelings include the following: feelings of uncertainty or anxiety over food (situation, resources, or supply); perceptions that food is of insufficient quantity (for adults and children); perceptions that food is of insufficient quality (includes aspects of dietary diversity, nutritional adequacy, preference); reported reductions of food intake (for adults and children); reported consequences of reduced food intake (for adults and children); and feelings of shame for resorting to socially unacceptable means to obtain food resources (Coates et al. 2007).

The HFIAS questionnaire consists of nine occurrence questions that represent a generally increasing level of severity of food insecurity (access) and nine “frequency‐of‐occurrence” questions that are asked as a follow‐up to each occurrence question to determine how often the condition occurred. The frequency‐of‐occurrence question is skipped if the respondent reports that the condition described in the corresponding occurrence question was not experienced in the previous 4 weeks (30 days) (Coates et al. 2007). The maximum score possible on the HFIAS is 27, which would represent the highest level of food insecurity.

### Agricultural biodiversity

No instrument to measure agricultural biodiversity had been developed and validated to the knowledge of the authors and hence a questionnaire was constructed using guidelines from FAO (2010) and a similar study in Kenya Ekesa et al. (2008). Agricultural biodiversity was measured by determining the variety of food plants grown, animals reared for food and food items obtained from natural habitats. A list of all food items grown, all animals reared, hunted, and other food items obtained from natural habitats through gathering or trapping was established for each household.

### Anthropometric measurements

The child health card, birth certificate, or baptism card were used to ascertain and record the age of the index child. In situations where the mother/care giver did not have the documents to ascertain the age of the child, they were asked to identify a child from the neighborhood who was born almost the same time. Children were excluded if they were younger than 24 months or older than 59 months.

*Height:* Children were measured in a standing position, using a free standing height/length stadiometer. Before taking the reading, the field worker ensured that the child was bare feet and that the heels, buttocks, shoulders, and the back of the head touched the stadiometer. Height was measured to the nearest 0.1 mm. Each of the measurements were taken twice and an average taken to ensure accuracy Gibson (2005).

#### Weight

Children were weighed on an electronic scale (Camry‐model EB9318) to the nearest 10 g. Children were weighed with minimum clothing, after emptying the bladder and without shoes (Gibson 2005). All the scales were calibrated by measuring a known weight to ensure that the correct measurement was achieved. Each of the measurements were taken twice and an average taken to ensure accuracy.

The children's anthropometric status was determined using the World Health Organization (2006) growth standards *Z*‐scores. Children with a *Z*‐score of less than ‐3SD for weight‐for‐age were categorized as being severely underweight, those with a *Z*‐score of −3 to −2SD were categorized as underweight, whereas those between −2 to −1SD were categorized as being mildly underweight or at risk of being underweight (World Health Organization 2006). Children with a *Z*‐score of less than −3SD for height‐for‐age were categorized as being severely stunted, those with a *Z*‐score of −3 to −2SD were categorized as stunted, whereas those between −2 and −1SD were categorized as mildly stunted or at risk of stunting. Children with a *Z*‐score <−3SD weight for‐height were categorized as being severely wasted, those with a *Z* score of −3 to −2 SD were categorized as wasted, whereas those between−2 and −1SD were categorized as mildly wasted or at risk of wasting.

### Data analysis

STATISTICA version 9 (StatSoft Inc. (2009) STATISTICA (data analysis software system)(www.statsoft.com), Statistical Package for Social Sciences (SPSS Version 11.5) and Food Finder 3 software were used to analyze the data collected by the 24 h recall. Dietary intake was measured during each season by conducting a repeated 24‐h recall with the mother/care giver of the household. A nutrient adequacy ratio (NAR) was calculated for each nutrient as the percent of the nutrient meeting the recommended dietary intake (RDI) value for that nutrient. A mean adequacy ratio (MAR) was calculated for 11 nutrients as the mean of the NARs of these nutrients. Dietary diversity was measured using data from the 24‐h recalls and classifying it into nine food groups. A DDS was calculated based on each of the food groups consumed during the period of recall up to a maximum of nine if foods had been consumed from each of the nine groups.

HFSwas measured using the HFIAS. HFIAS score was calculated for each household by summing the codes of each frequency‐of‐occurrence question. The agricultural biodiversity was calculated by counting the number of different crops and animals eaten either from domestic sources or from the wild. Weight and height measurements of children were taken. The following nutritional status indices were used to determine their nutritional status; Weight for age *Z* scores (WAZ), height for age *Z* scores (HAZ), and weight for height *Z* scores (WHZ) scores were determined for the children. The cut‐off was<− *Z* scores based on the WHO Child Growth Standards (2006). The relationships between continuous variables and nominal input variables were analyzed using appropriate analysis of variance (ANOVA) or pooled, paired, and independent mean *T*‐tests when only two groups were involved. When repeated measures were taken on the same respondents like the initial measurements and the 24‐h recall, it was done with the paired *t*‐test. For randomized designs the Mann–Whitney test or the Kruskal–Wallis test were used.

### Ethics

The study was approved by the Committee for Human Research, Faculty of Medicine and Health Sciences, Stellenbosch University (Reference No. N11/02/037) and each participant was required to sign a consent form. Thumb prints were used for participants who could not write. The researcher also obtained permission to conduct the research from the National Council for Science, Technology and Innovations (Kenya). Written informed consent was obtained from all subjects.

## Results

### Demographic and socioeconomic characteristics of the study population

Overall, the majority (87.8%) of the mothers/caregivers were married, 40.6% were casual laborers, 19.5% were homemakers, whereas 18.7% had no specific occupation (Table 1). About 10.1% of the mothers/care givers were petty traders, 5.4% were unemployed, 4.5% were self‐employed, and 1.2% were wage earners. The majority (84.6%) of mothers/care givers had attained a primary level of education, 5.0% had no formal education, 5.0% had some secondary education, and only 4.4% had completed secondary education. A significant difference was found to exist between the two divisions in the marital status (*P* < 0.001) and the occupation of the care giver (*P* < 0.001).

**Table 1:** Socio‐demographic characteristics of sample in Akithii and Uringu divisions of Kenya

Demographic and socioeconomic characteristics	AKITHII	URINGU	Total for both divisions	*χ* ^*2*^‐test*P*‐values
	*N*(%)	*N*(%)	*N*(%)	
Education of mother/care giver				
Primary	222 (85.4)	217 (83.8)	439 (84.6)	*χ *= 5.251*P* = 0.263
No formal education	16 (6.2)	10 (3.7)	26 (5.0)	
Some secondary	9 (3.5	17 (6.6)	26 (5.0)	
Completed secondary	10 (3.8	13 (5.0)	23 (4.4)	
Tertiary	3 (1.2)	2 (0.8)	5 (0.7)	
Marital status of mother/care giver				
Married	247 (94.3)	212 (81.2)	459 (87.8)	*χ *= 5.323*P* < 0.001*
Single	8 (3.1)	28 (10.7)	36 (6.9)	
Divorced/separated	1 (0.4)	14 (5.4)	15 (3.0)	
Widowed	6 (2.3)	7 (2.7)	13 (2.5)	
Occupation of mother/care giver				
Casual laborer	91 (35.0)	118 (46.5)	209 (40.7)	*χ *= 31.414*P* < 0.001*
Homemaker	49 (18.9)	51 (20.1)	100 (19.5)	
Petty trader	36 (13.8)	16 (6.3)	52 (10.1)	
Unemployed	7 (2.7)	21 (8.3)	28 (5.4)	
Self employed	15 (5.8)	8 (3.1)	23 (4.5)	
Wage‐earner	1 (0.4)	5 (2.0)	6 (1.2)	
Others	61 (23.5)	45 (13.8)	96 (18.7)	
Sources of drinking water				
Communal tap	183 (70.4)	30 (11.5)	213 (41.0)	*χ *= 208.454*P* < 0.0001*
River/lake/dam	49 (18.8)	138 (53.1)	187 (36.0)	
Well/borehole	17 (6.5)	86 (33.1)	103 (19.9)	
Own tap	11 (4.2)	3 (1.2)	14 (2.7)	
No. of rooms				
1–2 rooms	105 (40.4)	122 (46.9)	227 (43.7)	*χ *= 3.747*P* = 0.290
3–4 room	123 (47.3)	102 (39.2)	225 (43.3)	
5–6 rooms	27 (10.4)	32 (12.3)	59 (11.3)	
7 rooms above	5 (1.9)	4 (1.5)	9 (1.7)	
Cooking fuel				
Wood	249 (95.8)	248 (95.8)	497 (95.8)	*χ *= 1.511*P* = 0.825
Charcoal	6 (2.3)	4 (1.5)	10 (1.9)	
Gas	4 (1.5)	5 (1.9)	9 (1.7)	
Toilet type				
Pit	235 (92.2)	231 (89.9)	466 (91.0)	*χ *= 1.784*P* = 0.618
VIP	13 (5.1)	18 (7.0)	31 (6.1)	
None	7 (2.7)	7 (2.7)	14 (2.7)	
Decision on types food purchased				
Mother/care giver	171 (65.5)	141 (54.0)	312 (59.8)	*χ *= 17.590*P* = 0.007**
Husband/partner	78 (29.9)	87 (33.3)	165 (31.6)	
Grandmother/father	10 (3.8)	19 (7.3)	29 (5.6)	
Mother/In law	2 (0.8)	12 (4.6)	14 (2.7)	
Decision on the amount of money spent on food				
Husband/partner	133 (51.0)	130 (50.0)	263 (50.5)	*χ *= 20.997*P* = 0.013*
Mother/care giver	114 (43.7)	94 (36.2)	208 (39.9)	
Grandmother/father	10 (3.8)	18 (6.9)	28 (5.4)	
Mother/In law	2 (0.8)	10 (3.8)	12 (2.3)	
No. of people eating from the same pot				
1–2 persons	9 (3.5)	18 (6.9)	27 (5.2)	*χ *= 26.166*P* < 0.0001***
3–4 persons	62 (23.8)	100 (38.5))	162 (31.1)	
5–6 persons	111 (42.7)	102 (39.2)	213 (41.0)	
7–8 persons	67 (25.7)	31 (11.9)	98 (18.8)	
Above 9	11 (4.2)	9 (3.4)	20 (3.8)	
No. of contributors to h/hold income				
1 Person	108 (41.7)	99 (38.4)	207 (40.0)	*χ *= 5.501*P* = 0.240
2‐Person	143 (55.2)	145 (56.2)	288 (55.7)	
3–4 Person	5 (1.9)	11 (4.3)	16 (3.1)	
More than 5 person	3 (1.2)	3 (1.2)	6 (1.2)	
Amount of money spent on food weekly (Kes)				
0–800	18 (7.0)	37 (14.3)	55 (10.7)	*χ* = 37.381*P* < 0.0001***
801–1400	70 (27.2)	103 (39.9)	173 (33.6)	
1401–2000	56 (21.8)	59 (22.7)	115 (22.3)	
2001–2600	60 (23.3)	46 (17.8)	106 (20.6)	
2601 and above	53 (20.6)	13 (5.3)	65 (12.6)	

Significance* at *P* < 0.05; ***P < 0.001.

Forty‐one percent of households obtained their drinking water from communal taps, 36.0% from rivers, dams or lakes, whereas 19.8% consumed water from wells or boreholes. About half (43.7%) of the households had 1–2 and 43.3% had 3–4 roomed houses, whereas a very small percentage of 1.7% had houses with seven or more rooms. Most of the households had pit latrines (91.0%) and 6.1% with ventilated improved pit latrines, whereas 2.8% did not have toilets. The majority (95.8%) of the households used wood as fuel for cooking, whereas charcoal and gas were used by 1.9% and 1.7%, respectively. A significant difference was found to exist between the two divisions in the sources of water used (*P* < 0.001) with Akithii drinking mostly from communal taps and Uringu from rivers or wells.

In the majority of households, husbands/partners (50.5%), followed by mothers/caregivers (39.9%) made the decision on how much money was spent on food. Most households in both divisions had 3–4 or 5–6 persons eating from the same pot. The number of persons contributing to household income in the slightly over half (55.7%) of households were two persons, followed by one person (40.0%) and three to four persons (3.1%). About 22.3% of the households spent Kes 1401 to Kes 2000 (~$23.3) per week on food.

A significant difference was found to exist between the two divisions in the decision on the types of food to be purchased for a household (*P* = 0.007), the decision on the amount of money to be spent on food (*P* = 0.013), and on the amount of money spent on a weekly basis on purchasing food (*P* < 0.0001).

### Anthropometric status of children 24–59 months old

Overall the percentage of children having a HAZ <−2SD, HAZ −2SD to −3SD, and HAZ <−3SD were lower in Akithii than in Uringu (Table 2). The mean HAZ scores were −1.46 and −1.44 in Akithii and −1.29 and −1.29 in Uringu during which phase 1 and 2 of data collection, respectively. The percentage of children with stunted growth (HAZ<−2SD) was 34.7 and 31.9% in Akithii for phase 1 and 2, respectively, and 26.3% and 28.2% in Uringu. The percentage of children with a WAZ <−2SD, WAZ −2 to −3SD and WAZ <−3SD were lower in Akithii than in Uringu. The mean WAZ scores were −1.19 and −1.13 in Akithii and −1.04 and −1.09 in Uringu. The percentage of children who were underweight (WAZ<−2SD) was 21.6% and 16.7% in Akithii for phases 1 and 2, respectively, and 20.3% and 20.2% in Uringu for phases 1 and 2, respectively. Overall the percentage of children having a WHZ <−2SD, WHZ −2 to −3SD, and WHZ <−3SD were similar in Akithii and Uringu. The mean WHZ scores were −0.52 and −0.43 in Akithii and −0.45 and −0.53 in Uringu for phase 1 and 2, respectively. The percentage of children with wasting (WHZ<−2SD was 7.9% and 4.6% in Akithii for phase 1 and 2, respectively, and 5.7% and 8.8% in Uringu, respectively. There were no significant differences in anthropometric variables between phases or between the two areas.

**Table 2:** Percentage of children in the sample in Kenya at phases 1 and 2 of the study having anthropometric values below cut‐off values

Anthropometric indices	Akithii phase 1(*n* = 245)% (95%CI)	Akithii phase 2(*n* = 245)% (95%CI)	Uringu phase 1(*N* = 232)% (95%CI)	Uringu phase 2(*n* = 232)% (95%CI)
WHZ<−2 D	7.9 (5.2–11.8)	4.6 (2.4–8.5)	5.7 (3.4–9.4)	8.8 (5.8–13.1)
WHZ−2 to −3SD	7.1 (4.5–10.9)	4.2 (2.2–7.8)	3.3 (1.7–6.3)	8.8 (5.8–13.1)
WHZ<−3SD	0.8 (0.2–2.8)	0.4 (0.2–0.8)	2.4 (1.1–5.2)	0.0 (0.0–1.6)
Mean WHZ95% CI	−0.52 (−0.7 to −0.4)	−0.43 (−0.6–0.3)	−0.45 (−0.6 to −0.3)	−0.53 (−0.7 to −0.4)
WAZ <−2SD	21.6 (16.9–27.2)	16.7 (8.5–30.2)	20.315.6–25.9	20.2 (15.6–25.7)
WAZ<−2 to −3SD	17.1 (12.9–22.4)	11.3 (5.8–20.7)	6.0 (3.6–9.9)	16.8 (12.6–22.1)
WAZ<−3SD	4.5 (2.5–7.9)	5.4% (2.8–10.1)	3.4 (1.7–6.6)	3.4 (1.7–6.5)
Mean WAZ95% CI	−1.19 (−1.3 to −1.3)	−1.13 (−1.3 to −1.0)	−1.04 (−1.2 to −1.1)	−1.09 (−1.2 to −1.0)
HAZ <−2SD	34.7 (29.0–40.8)	31.9 (23.4–41.7)	26.3 (21.0–32.3)	28.2 (22.8–34.2)
HAZ −2 to −3SD	25.7 (20.6–31.5)	22.3 (19.1–25.9)	20.3 (15.6–25.9)	21.4 (16.7–7.1)
HAZ<−3SD	9.0 (6.0–13.2)	9.6 (5.1–17.1)	6.0 (3.6–9.9)	6.7 (4.2–10.6)
Mean HAZ95% CI	−1.46 (−1.6– −1.3)	−1.44 (−1.6 to−1.3)	−1.29 (−1.4 to −1.1)	−1.29 (−1.5 to −1.1)

There was a positive significant relationship (*P* = 0.0001) between the number of those contributing to household income and weight status (WAZ) (Table 3). Furthermore, both WHZ (*P* = 0.04) and WAZ (*P* = 0.02) were associated with the amount of money spent on food. The number of people eating from the same pot was negatively associated with WAZ (*P* = −0.03) implying that as number of people increased weight decreased.

**Table 3:** Associations between socio‐demographic characteristics with child nutritional status in both phases in both divisions

Socio‐demographic characteristics	WHZ	WAZ	HAZ
No. of contributors to household income	Kruskal–Wallis *P* = 0.38	Kruskal–Wallis *P* = 0.001***	Kruskal–Wallis *P* = 0.14
Amount of money spent on food per week	Kruskal–Wallis *P* = 0.04*	Kruskal–Wallis *P* = 0.02*	Kruskal–Wallis *P* = 0.55
No. of people eating from the same pot	Spearman *r* = −0.06, *P* = 0.17	Spearman *r* = −0.10, *P* = 0.03*	Spearman *r* = −0.08, *P* = 0.08

WHZ, weight for height *Z* score; WAZ, weight for age *Z* score; HAZ, height for age *Z* score.

Significance* at *P* < 0.05; ***P < 0.001.

### Dietary diversity, household food inventory assess scale, and agricultural biodiversity

Table 4 presents data on DDS, HFIAS, and AB. From this we note that about 30% more children in Akithii had a poor DDS (<4 groups) compared with Uringu (79.7% vs. 51.6%). Mean DDS in Akithii (2.9) was significantly lower than Uringu (3.7) at both phases (*P* < 0.0001). Akithii also had a significantly higher mean HFIAS (16.2 and 12.5) than Uringu (10.0 and 9.3) indicative of poorer food security(*P* < 0.0001).The percentage of households with severe food insecurity was 82% and 58% in Akithii in phases 1 and 2, respectively, compared with 47% and 42% in Uringu, respectively. Mean AB was, also significantly lower in Akithii compared with Uringu (6.6 vs. 7.2 items), respectively.

**Table 4:** Mean DDS, mean HFIAS, mean AB in each division over the two phases of the study

Variables	Akithii phase 1 (*n* = 245)	Akithii phase 2 (*n* = 245)	Uringu phase 1 (*N* = 232)	Uringu phase 2 (*N* = 232)	Independent *T*‐test between divisions phase 1	Independent *T*‐test between divisions phase 2
Mean DDS	2.9	2.9	3.7	3.7	*P* < 0.0001	*P* < 0.0001
SD	1.10	1.0	1.12	1.11		
DDS < 4	79.7%	79.7%	51.6%	52.3%		
Mean HFIAS	16.2	12.5	10.0	9.3	*P* < 0.0001	*P* < 0.0001
SD	7.01	7.80	6.90	7.02		
Food secure	2%	11%	12%	18%		
Mild HFIS	1%	4%	7%	6%		
Moderate HFIS	14%	27%	33%	33%		
Severe HFIS	82%	58%	47%	42%		
Mean AB	6.6%	N/A	7.2	N/A	*P* = 0.035	N/A
SD	2.44	N/A	4.19	N/A		

DDS, dietary diversity; HFIAS, household food insecurity access scale; HFIS, household food insecurity; AB, agricultural biodiversity; SD, standard deviation; N/A, AB not measured in Phase 2.

### Relationship between dietary diversity, agricultural biodiversity, household food security, and child anthropometric status

Child anthropometric indices (WAZ, HAZ, and WHZ) were correlated with DDS, AB, and HFIAS to determine the relationships between these variables (Table 5). No significant correlation was found to exist between WAZ and WHZ with DDS in this study. There was, however, a significant relationship between stunting in children (HAZ<−2SD) and dietary diversity in Phase 2 data. An increase in DDS appeared to reflect a decrease in stunting in children. No significant relationship was found to exist between AB with WAZ, HAZ, or WHZ scores or with HFIAS with WAZ, HAZ, or WHZ scores.

**Table 5:** Association of DDS, AB, and HFIAS with each other and anthropometric variables

Variables	Spearman *R*	*T*(*n*–2)	*P* value
Phase 1 DDS			
DDS & WAZ	−0.015	−0.335	0.73
DDS & HAZ	−0.005	−0.120	0.90
DDS & WHZ	−0.009	−0.202	0.83
Phase 2 DDS			
DDS & WAZ	0.068	1.439	0.150
DDS & HAZ	0.114	2.455	0.014
DDS & WHZ	0.021	0.454	0.649
Phase 1			
AB & WAZ	0.047	1.009	0.313
AB & HAZ	−0.005	−0.114	0.909
AB & WHZ	0.079	1.726	0.085
Phase 2			
AB & WAZ	−0.036	−0.544	0.586
AB & HAZ	−0.087	−1.311	0.191
AB & WHZ	0.088	1.359	0.175
HFIAS both phases	Pearson		
HFIAS & WAZ	−0.048	884	0.156
HFIAS & HAZ	−0.037	894	0.264
HFIAS & WHZ	−0.031	906	0.352
DDS with HFIAS	During Phase 1, the correlation using Spearman *R* was (*R* = −0.158, *t* (*N*–2) −3.565), *P* = 0.0003), whereas during Phase 2 the correlation was (*R* = −0.185, *t* (*N*–2) −3.889), *P* = 0.0001). This correlation shows that an increase in dietary diversity inversely affected HFIAS.		
AB &HFIAS	Households with higher AB more likely to be food secure; Spearman *r* = −0.136, *P* = 0.002		
AB & DDS	Households with a high AB were likely to have a high DDS; ANOVA *F*(1,496) = 14.791, *P* = 0.00014.		

DDS, dietary diversity; HFIAS, household food insecurity access scale; AB, agricultural biodiversity; DS, dietary diversity; HFIAS, household food insecurity access scale; HFI, household food insecurity; AB, agricultural biodiversity; SD, standard deviation; N/A, AB not measured in Phase 2.

Table 5 also shows the relationship between HFIAS with DDS and AB. In both instances there is a significant inverse relationship (*P* < 0.01). Higher DDS and AB scores lead to better household food security. There is also a significant association between AB and DDS; as AB increases, so does DDS (*P* = 0.00014).

### Relating dietary diversity, agricultural biodiversity, and household food security in households with and without children with stunted growth

The relationship between DDS, agricultural biodiversity score, and HFIAS in households with and without children with stunted growth was investigated. During Phase 1, there was a significant difference between households with and without children with stunted growth for the variable mean DDS (*P* = 0.047) and also for the variable mean HFIAS (*P* = 0.009) in phase 2 (Table 6). Agricultural biodiversity did not show any significant differences between the two groups of households. The means for households with children presenting with stunted growth were, however, lower at 6.8, as compared to 7.0 of those with normal growth.

**Table 6:** Relating dietary diversity, agricultural biodiversity, and household food security in households with and without children with stunted growth in both phases and divisions

Variables	Divisions		Households with children without Stunted growth	Households with children with Stunted growth	ANOVA, *P*‐value
DDS	Akithii &UringuPhase 1	Mean	3.3 (SD,1.41)	3.3 (SD, 1.22)	*P* = 0.651
		*N*	314	139	
	Akithii &UringuPhase 2	Mean	3.3 (SD,1.12)	3.1 (SD, 1.13)	*P* = 0.047*
		*N*	317	136	
	Both groups(Phase 1& 2)	Mean	3.3 (SD, 1.13)	3.2 (SD, 1.18)	*P* = 0.090
		*N*	*N* = 631	*N* = 275	
HFIAS	Akithii &UringuPhase 1	Mean	12.4 (SD,7.38)	14.3 (SD, 7.17)	*P* = 0.009*
		*N*	331	145	
	Akithii &UringuPhase 2	Mean	11.1	10.1	*P* = 0.232
		*N*	291	127	
	Both groups (Phase 1& 2)	Mean	11.8 (SD,7.61)	12.4 (SD, 7.45)	*P* = 0.310
		*N*	*N* = 622	*N* = 272	
AB	Both groups	Mean	7.0 (SD, 3.14)	6.8 (SD, 3.45)	*P* = 0.486
		*N*	*N* = 317	*N* = 142	

Significance*at *P* < 0.05.

## Discussion

In general, the prevalence of undernutrition in the study area can be classified as medium to high according to WHO standards (1997). There were marked differences in the prevalence of undernutrition in the two study areas across the two phases of data collection with Akithii being classified as an area with a high level of undernutrition, whereas Uringu was classified as presenting with a medium level of undernutrition. Uringu had a higher level of household assets (probably implying higher socioeconomic status), DDS and AB compared to Akithii may be contributing the lower undernutrition levels in this region. Akithii had higher percentages of children who presented with underweight and wasting compared to the national levels in Kenya (Kenya National Bureau of Statistics (KNBS) and ICF Macro 2010). The level of stunting was, however, the same as the national level at 35% (GOK 2009a; Kenya National Bureau of Statistics (KNBS) and ICF Macro, 2010) Uringu, in contrast, had a lower level of child undernutrition compared to the national rate. During phase 2 of the study, the percentages of children who presented with wasting, underweight, and stunting in both the study sites were generally lower compared to phase 1. This may be attributed to the effect of seasonality. During phase 2 of the study, household food security and dietary diversity improved, probably because it was during the rainy reason, unlike during phase 1 when data collection took place during the dry season.

Socio‐demographic characteristics that may affect vulnerability to food insecurity and anthropometric status in children were investigated. In this study, a significant positive relationship was found between the number of contributors to household income and WAZ scores of children. The amount of money spent on food per week significantly affected WAZ and WHZ but not HAZ values. A significant inverse relationship was also found between the number of people eating from the same pot and WAZ scores, namely, the higher the number of people eating from the same pot, the lower the WAZ values. The number of assets owned by a household also significantly influenced HAZ, scores; the higher the numbers of household assets, the higher the HAZ values were. It is important to note that the households in Akithii which is a semiarid region, (GOK 2002, 2009b) spent more money on food on a weekly basis compared to Uringu which has a high agricultural productivity (GOK 2002, 2009b)

Despite evidence from other studies showing associations between WAZ and WHZ with DDS, (Arimond and Ruel 2004; Sawadogo et al. 2006; Ekesa et al. 2008) few significant relationships were found in this study. Another recent study in Kenya also found no significant relationship between DDS, WAZ, and WHZ, (Nungo et al. 2012).^.^ There was, however, a significant positive relationship in this study between stunting in children (HAZ) with dietary diversity of the child in phase 2 of the study. A higher DDS was associated with lower stunting levels. This relationship signifies that households that had the dietary diversity score plays a significant role in child nutrition, especially HAZ. The latter finding concurs with a study in rural Bangladesh which found that reduced dietary diversity was a strong predictor of stunting in children aged 6–59 months. High dietary diversity was associated with a 31% reduced odds of being stunted among children 24–59 months, after adjusting for all potential confounders (Rah et al. 2010). Findings of other studies suggest that there is an association between child dietary diversity and nutritional status that is independent of socioeconomic factors and that dietary diversity may indeed reflect diet quality (Sawadogo et al. 2006; Penafiel et al. 2011)

In this study, there was no significant relationship between household food security and child nutritional status based on anthropometric measurements. This implies that the food security situation of the households was not related with the nutritional status of the children. While some studies have reported a positive association between household food insecurity and childhood growth indicators, (Melgar‐Quinonez et al. 2009; Chaparro 2012) others have found no relationship, (Alaimo et al. 2001; Akoto et al. 2010; Kac et al. 2012). The lack of association between nutritional status indicators and the food security indicator is difficult to explain, but suggests that the relationships are more complex due to the multifaceted nature of these variables.

Due to the multifaceted nature of malnutrition, Ruel and Alderman (2013) argues that food security is necessary but not sufficient to ensure adequate nutrition and to prevent malnutrition in children because care givers also need to provide them with care, hygiene, and health‐seeking practices in order for them to grow, develop, and remain healthy. Pinstrup‐Andersen (2013)also emphasizes that even if household food security is achieved, malnutrition may flourish due to intrahousehold distribution, which may not correspond to individual needs or because nonfood factors that are important for nutrition such as unclean water, poor sanitation, and hygiene and inappropriate care are the most serious constraints to good nutrition. This could probably explain the lack of association between household food security status and child nutritional status based on WAZ and WHZ in this study.

Household food security in this study was significantly associated with DDS and AB, implying that a diet high in dietary diversity and agricultural biodiversity improves household food security. Dietary diversity also showed a significant positive association with agricultural biodiversity. Kenya has been described as a country rich in agricultural biodiversity with an estimated 35,000 known species of animals, plants, and microorganisms (GOK 2001). The country's agricultural biodiversity is, however, under serious threat due to among others increasing deforestation, climate change, pollution, and soil degradation (Alaimo et al. 2001). The level of agricultural biodiversity (*n* = 26) in Tigania west in the Eastern part of Kenya, the area of study, was found to be low and far less than the number described in an earlier study conducted in western Kenya which identified 41 different species of food in the natural habitat (Pelletier and Frongillo 2003). The improvement of agricultural biodiversity is key in ensuring diverse diets and in cushioning households from climatic shocks caused by changing weather patterns since most households depends on rain‐fed agriculture. Food‐based strategies have been recommended as the first priority to meet micronutrient needs (Allen 2008). A more diverse diet holds the potential to provide a more abundant supply of both macro‐ and micronutrients and could therefore be one of the approaches to ensure greater food and nutrition security in Kenya.

A significant difference was established between households with and without children with stunted growth in DDS during the rainy season and HFIAS in the dry season, but not with the AB score. The mean AB for households with children with stunted growth were lower compared to those with normal growth, but did not reach significant level. This suggests that DDS and household food security may have the potential of being used as proxy measures for stunting. The results of this study concur with results from an analysis based on data from five developing countries which included Bangladesh, Nepal, Pakistan, Tanzania, and Uganda (Tiwari et al. 2013). These results demonstrated that various measures of household food security significantly correlated with the nutritional status of children within a household. These results were significant even when the socioeconomic characteristics of the households were controlled. The analysis concluded that food security indicators such as food consumption and DDS can be used as proxy measures for the underlying nutritional status of children (Tiwari et al. 2013).

## Conclusion

Households that have children with and without stunted growth were significantly different in individual dietary diversity and household food security levels in the dry (shortage season = Phase 1) and rainy season (after harvest season Phase 2), but not with regard to agricultural biodiversity scores. DDS influences HAZ and HFIAS, whereas households with a higher agricultural biodiversity (AB) had higher DDS. Households with a higher AB were more food secure compared to those with lower AB. DDS, HFS, and AB played a significant role in determining the levels stunting of children among children of 24–59 months. This suggests some potential in using DDS and HFIAS as proxy measures for stunting. The Interventions to improve child nutritional status in resource‐poor rural households such as in the study area should therefore aim at increasing dietary diversity and/or AB in order to improve household food security.

## Ethical Standards Disclosure

This study was conducted according to the guidelines laid down in the Declaration of Helsinki and all procedures involving human subjects were approved by Committee for Human Research, Faculty of Medicine and Health Sciences, Stellenbosch University (ethics reference no.N11/02/037).

## Conflict of Interest

The authors declare that there is no conflict of interest.
